# Opportunities to Improve Communication With Residency Applicants: Cross-Sectional Study of Obstetrics and Gynecology Residency Program Websites

**DOI:** 10.2196/48518

**Published:** 2024-10-21

**Authors:** Paulina M Devlin, Oluwabukola Akingbola, Jody Stonehocker, James T Fitzgerald, Abigail Ford Winkel, Maya M Hammoud, Helen K Morgan

**Affiliations:** 1 Department of Obstetrics and Gynecology, University of Utah Health, Salt Lake City, UT, United States; 2 Department of Obstetrics, Gynecology and Women's Health, University of Minnesota Medical School, Minneapolis, MN, United States; 3 Department of Obstetrics and Gynecology, University of New Mexico, Albuquerque, NM, United States; 4 Department of Learning Health Sciences, University of Michigan, 1500 E Medical Center Dr, Ann Arbor, MI, 48109, United States, 1 734-936-3110, 1 734-936-7722; 5 Department of Obstetrics and Gynecology, New York University Grossman School of Medicine, New York, NY, United States; 6 Department of Obstetrics and Gynecology, University of Michigan, Ann Arbor, MI, United States

**Keywords:** obstetrics and gynecology, residency program, residency application, website, program signals, communication best practices

## Abstract

**Background:**

As part of the residency application process in the United States, many medical specialties now offer applicants the opportunity to send program signals that indicate high interest to a limited number of residency programs. To determine which residency programs to apply to, and which programs to send signals to, applicants need accurate information to determine which programs align with their future training goals. Most applicants use a program’s website to review program characteristics and criteria, so describing the current state of residency program websites can inform programs of best practices.

**Objective:**

This study aims to characterize information available on obstetrics and gynecology residency program websites and to determine whether there are differences in information available between different types of residency programs.

**Methods:**

This was a cross-sectional observational study of all US obstetrics and gynecology residency program website content. The authorship group identified factors that would be useful for residency applicants around program demographics and learner trajectories; application criteria including standardized testing metrics, residency statistics, and benefits; and diversity, equity, and inclusion mission statements and values. Two authors examined all available websites from November 2011 through March 2022. Data analysis consisted of descriptive statistics and one-way ANOVA, with *P*<.05 considered significant.

**Results:**

Among 290 programs, 283 (97.6%) had websites; 238 (82.1%) listed medical schools of current residents; 158 (54.5%) described residency alumni trajectories; 107 (36.9%) included guidance related to the preferred United States Medical Licensing Examination Step 1 scores; 53 (18.3%) included guidance related to the Comprehensive Osteopathic Medical Licensing Examination Level 1 scores; 185 (63.8%) included international applicant guidance; 132 (45.5%) included a program-specific mission statement; 84 (29%) included a diversity, equity, and inclusion statement; and 167 (57.6%) included program-specific media or links to program social media on their websites. University-based programs were more likely to include a variety of information compared to community-based university-affiliated and community-based programs, including medical schools of current residents (113/123, 91.9%, university-based; 85/111, 76.6%, community-based university-affiliated; 40/56, 71.4%, community-based; *P*<.001); alumni trajectories (90/123, 73.2%, university-based; 51/111, 45.9%, community-based university-affiliated; 17/56, 30.4%, community-based; *P*<.001); the United States Medical Licensing Examination Step 1 score guidance (58/123, 47.2%, university-based; 36/111, 32.4%, community-based university-affiliated; 13/56, 23.2%, community-based; *P*=.004); and diversity, equity, and inclusion statements (57/123, 46.3%, university-based; 19/111, 17.1%, community-based university-affiliated; 8/56, 14.3%, community-based; *P*<.001).

**Conclusions:**

There are opportunities to improve the quantity and quality of data on residency websites. From this work, we propose best practices for what information should be included on residency websites that will enable applicants to make informed decisions.

## Introduction

In the United States, becoming an accredited physician is a rigorous and competitive process where candidates complete undergraduate training, medical school education, and residency training in a chosen specialty. Typically, individuals first obtain an undergraduate degree to gain admittance to a medical school. Next, they must earn a medical doctorate (MD) or doctor of osteopathic medicine (DO) from an accredited medical school or an equivalent international medical degree. Finally, they must complete postgraduate residency training; to fulfill this requirement, individuals apply to a residency program in their intended specialty. In the United States, many residency applicants are medical students in their final year of training, but individuals may also apply if they previously completed an MD or DO degree or completed medical school outside the United States and obtained certification from the Educational Commission for Foreign Medical Graduates [1]. All residency programs fulfill requirements set by the Accreditation Council for Graduate Medical Education, but programs have different strengths. Residency programs may be based in large university academic centers, community medical centers, or medical centers in a community setting that are affiliated with universities and often consequently emphasize clinical service to communities versus academic pursuits in training. Applying for residency is a competitive step in the physician training process; qualified applicants often apply to programs in a matching system that algorithmically matches applicants into programs that rank the applicant. In 2022, a total of 42,549 applicants were matched into 36,943 residency positions in the National Resident Matching Program Main Residency Match, making the overall match rate for all active applicants 86.8% [2]. This match rate, however, does not illustrate the full story; there is a wide range of match rates for different types of applicants and specialties, and the number of applicants who do not match into their top programs of interest is increasing [3].

Due to this competitiveness, now more than ever, residency applicants need transparent data to make informed decisions during the residency application process. Applicants determine where to apply, and among an increasing number of specialties, they must also decide where to send program signals—electronic tokens indicating high interest in a program—at the time of application submission. In the 2022‐2023 application cycle, 17 specialties opted to include program signaling [4-7]. Ideally, applicants should apply and send signals to programs that align with their values and priorities and to programs where they have a reasonable chance of matching [4]. Determining which programs meet these criteria is a challenge for applicants; they rely on a variety of nationally available data sources [8,9] and have particularly valued information from program websites for their application decision-making [10-12]. Therefore, our study sought to characterize content available on obstetrics and gynecology (OBGYN) residency program websites and to determine whether there were differences in website content according to program type and geographic location. Our goal was to use this information to inform best practices for residency program websites.

## Methods

### Study Design

This was a cross-sectional observational study of US OBGYN residency program websites. We examined programs listed on the Electronic Residency Application Service (ERAS) 2022 Participating Specialties and Programs website. All programs listed on March 22, 2022, were included. Data for whether the type or program was university-based, community-based university-affiliated, or community-based were obtained by searching for the program in the American Medical Association’s Fellowship and Residency Electronic Interactive Database Access System. Data for the census region and division of programs were determined based on the US Census Bureau Regions and Divisions with State FIPS Codes document.

Two authors (PMD and OA) collected data between November 2021 and March 2022. After obtaining the list of residency programs, we searched for a website associated with the program through a direct link from the ERAS list. In cases where a link was unavailable or incorrect, a Google search was conducted to attempt to find a website. Individual programs were not contacted directly by the study team.

The authorship group identified factors that would be useful for residency applicants. This group consisted of OBGYN faculty with education leadership roles, an OBGYN resident, and an OBGYN medical student applicant. The group used experiences from these roles to iteratively create a list of factors to consider, including program demographics and learner trajectories, application criteria including standardized testing metrics, residency salary and benefits, and diversity, equity, and inclusion mission statements and values. Variables described whether particular information was available on websites and were classified as yes or no. Variable information needed to be available on the program website and its website pages, or via a direct link from the program website and pages. Each website page linked from the main page of the residency website was reviewed for content, and direct links that were judged likely to be relevant were also opened. Data were entered in a Google spreadsheet for collection. In cases of ambiguity, PMD and OA discussed the content and agreed on the determination. To confirm accuracy and interrater reliability, after completing data collection, 10% of records as determined by random number generation were checked, with no systematic errors identified. Interrater reliability was not formally calculated; however, a few data entries were incongruent. All collected variables are described in Table 1.

**Table 1.** Content of obstetrics and gynecology residency program websites and comparison by type of residency program (N=290).

Characteristic		Total programs,n (%)	U^a^ programs, n (%)	CU^b^ programs, n (%)	C^c^ programs, n (%)	ANOVA*, P* value	Post hoc comparisons, global^d^
Website		283 (97.6)	123 (100)	108 (97.3)	52 (92.9)	.02	U>C
Medical schools of residents		238 (82.1)	113 (91.9)	85 (76.6)	40 (71.4)	<.001	U>CU and U>C
Alumni trajectories		158 (54.5)	90 (73.2)	51 (45.9)	17 (30.4)	<.001	U>CU and U>C
**USMLE^e^ requirements**		225 (77.6)	108 (87.8)	77 (69.4)	40 (71.4)	.001	U>CU and U>C
	Step 1 attempts considered	77 (26.6)	29 (23.6)	26 (23.4)	22 (39.3)	.06	N/A^f^
	Step 1 program notes no minimum noted	48 (16.6)	36 (29.3)	10 (9.0)	2 (3.6)	<.001	U>CU and U>C
	Step 1 range, averages, or suggestions other than passing or no minimum	64 (22.1)	26 (21.1)	27 (24.3)	11 (19.6)	.75	N/A
	Step 1 any score guidance other than passing	107 (36.9)	58 (47.2)	36 (32.4)	13 (23.2)	.004	U>CU and U>C
**COMLEX^g^ requirements**		143 (49.3)	52 (42.3)	57 (51.4)	34 (60.7)	.06	N/A
	Level 1 attempts considered	39 (13.4)	9 (7.3)	16 (14.4)	14 (25.0)	.005	C>U
	Level 1 program notes no minimum noted	16 (5.5)	8 (6.5)	7 (6.3)	1 (1.8)	.40	N/A
	Level 1 range, averages, or suggestions other than passing or no minimum	36 (12.4)	9 (7.3)	17 (15.3)	10 (17.9)	.07	N/A
	Level 1 any score guidance other than passing	53 (18.3)	17 (13.8)	24 (21.6)	12 (21.4)	.24	N/A
Discusses DACA^h^ applicants		0 (0.0)	0 (0.0)	0 (0.0)	0 (0.0)	N/A	N/A
Indication of whether international applicants are considered^i^		185 (63.8)	93 (75.6)	57 (51.4)	35 (62.5)	<.001	U>CU
Residency mission statement		132 (45.5)	61 (49.6)	51 (45.9)	20 (35.7)	.23	N/A
Residency diversity, equity, and inclusion statement or link to departmental statement		84 (29.0)	57 (46.3)	19 (17.1)	8 (14.3)	<.001	U>CU and U>C
Fellowship availability noted or directly accessible from residency website		128 (44.1)	96 (78.0)	27 (24.3)	5 (8.9)	<.001	U>CU and U>C
Average or estimated number of applications disclosed		23 (7.9)	11 (8.9)	10 (9.0)	2 (3.6)	.41	N/A
Average or estimated interview invitations disclosed		23 (7.9)	17 (13.8)	6 (5.4)	0 (0.0)	.003	U>CU and U>C
Salary noted or direct link to salary		185 (63.8)	80 (65.0)	65 (58.6)	40 (71.4)	.25	N/A
Benefits noted or direct link to benefits		200 (69.0)	89 (72.4)	66 (59.5)	45 (80.4)	.01	C>CU
Rotations according to residency year noted		248 (85.5)	111 (90.2)	90 (81.1)	47 (83.9)	.13	N/A
Indication of average or most recent ACGME^j^ case numbers per resident		39 (13.4)	18 (14.6)	18 (16.2)	3 (5.4)	.13	N/A
Program-specific videos or links to social media		167 (57.6)	80 (65.0)	62 (55.9)	25 (44.6)	.03	U>C

a U: university-based.

b CU: community-based university-affiliated.

c C: community-based.

d *P*=.05.

e USMLE: United States Medical Licensing Examination.

f N/A: not applicable.

g COMLEX: Comprehensive Osteopathic Medical Licensing Examination of the United States.

h DACA: Deferred Action for Childhood Arrivals.

i Including discussion on visa sponsorship.

j ACGME: Accreditation Council for Graduate Medical Education.

Data were exported from the Google spreadsheet as an .xlsx file and uploaded into JMP Pro 17.0.0 (SAS Institute, Inc), which was used to conduct statistical analysis. Descriptive statistics and one-way ANOVA were performed to determine differences among the three types of programs using a significance level of .05. Post hoc comparisons used the Tukey-Kramer honest significant difference (global *P*=.05).

### Ethical Considerations

This study was considered by the University of Michigan's IRBMED institutional review board (study identification HUM00218409). The board determined that, in accordance with the board and federal regulations, the study did not require institutional review board approval because it considered publicly available data that could not be identified with a human subject.

## Results

Of 290 OBGYN residency programs, 123 (42.4%) were university-based programs, 111 (38.3%) were community-based university-affiliated, and 56 (19%) were community-based. Most programs (283/290, 97.6%) had websites. Many programs did not include information about whether standardized testing filtering metrics are applied to applications (details are in Table 1). Notably, less than half (143/290, 49.3%) included any information about the Comprehensive Osteopathic Medical Licensing Examination (COMLEX). A majority of programs (238/290, 82.1%) listed the medical school of current residents, but fewer (158/290, 54.5%) described alumni trajectories. No programs discussed whether applicants with Deferred Action for Childhood Arrivals status would be considered.

When comparing types of programs, university-based programs were more likely to include a variety of information on their websites compared to community-based university-affiliated programs and community-based programs, including medical schools of current residents (113/123, 91.9%, university-based; 85/111, 76.6%, community-based university-affiliated; 40/56, 71.4%, community-based; *P*<.001); alumni trajectories (90/123, 73.2%, university-based; 51/111, 45.9%, community-based university-affiliated; 17/56, 30.4%, community-based; *P*<.001); statements about whether the United States Medical Licensing Examination (USMLE) Step 1 is required (108/123, 87.8%, university-based; 77/111, 69.4%, community-based university-affiliated; 40/56, 71.4%, community-based; *P=*.001); statements about no minimum USMLE score (36/123, 29.3%, university-based; 10/111, 9%, community-based university-affiliated; 2/56, 3.6%, community-based; *P<*.001); any USMLE score guidance other than a passing grade (58/123, 47.2%, university-based; 36/111, 32.4%, community-based university-affiliated; 13/56, 23.2%, community-based; *P*=.004); diversity, equity, and inclusion statements (57/123, 46.3%, university-based; 19/111, 17.1%, community-based university-affiliated; 8/56, 14.3%, community-based; *P*<.001); discussion of availability of fellowships at the same institution (96/123, 78%, university-based; 27/111, 24.3%, community-based university-affiliated; 5/56, 8.9%, community-based; *P<*.001); and whether the average or estimated number of interview invitations were disclosed (17/123, 13.8%, university-based; 6/111, 5.4%, community-based university-affiliated; 0/56, 0%, community-based; *P=*.003).

On post hoc analysis, there were several characteristics with overall significantly different representation on the websites of different types of programs but not between all types of programs. On post hoc comparison, university-based programs had websites significantly more often than community-based programs, but not significantly more often than community-based university-affiliated programs (123/123, 100%, university-based; 108/111, 97.3%, community-based university-affiliated; 52/56, 92.9%, community-based; *P=*.02). University-based program websites indicated whether international applicants were considered significantly more often than community-based university-affiliated programs, but not significantly more often than community-based programs (93/123, 75.6%, university-based; 57/111, 51.4%, community-based university-affiliated; 35/56, 62.5%, community-based; *P<*.001). University-based program websites had significantly more program-specific videos or links to social media than community-based programs, but not community-based university-affiliated programs (80/123, 65%, university-based; 62/111, 55.9%, community-based university-affiliated; 25/56, 44.6%, community-based; *P=*.03).

Additionally, on post hoc comparison of significant findings, two of the 25 characteristics studied had a different pattern of presence on program websites. Community-based program websites noted whether COMLEX Level 1 attempts were considered significantly more often than university-based program websites, but not more often than community-based-university affiliated programs (9/123, 7.3%, university-based; 16/111, 14.4%, community-based university-affiliated; 14/56, 25%, community-based; *P=*.005), and community-based program websites noted benefits or directly linked to benefits significantly more often than community-based university-affiliated programs, but not more often than university-based programs (89/123, 72.4%, university-based; 66/111, 59.5%, community-based university-affiliated; 45/56, 80.4%, community-based; *P=*.01). Further description is listed in Table 1. There were minimal differences based on geographic location.

## Discussion

### Principal Results

Many OBGYN residency program websites lack information that is necessary for applicants to make informed decisions about where to apply and send program signals. When comparing types of programs, we found significant differences in website content, with many factors more often included by university-based programs than by community-based university-affiliated and community-based programs. Although this study was limited to OBGYN, these findings are relevant to all specialties, especially given the need for multiple intervention points for widespread residency application reform [3].

At this important educational transition point, applicants should ideally select residency programs that will enable them to thrive, both personally and professionally, during and after residency training. Many factors should be considered in learners’ self-reflection processes, including whether they want to practice in an academic or community setting, their goals for research and fellowship training, and their individual learning styles. For residency programs to facilitate this decision-making process, this information should be available on program websites, particularly given applicants’ reliance on this source [10-12]. Our work suggests that community-based university-affiliated programs and community-based programs currently lag behind university-based programs in several factors on their websites; consequently, applicants may miss an opportunity to learn about whether these programs align with their needs.

Our work is particularly salient given the widespread adoption of program signaling by many specialties. Transparency around application criteria is necessary if this meaningful residency application reform is to be successful. Notably, detailed standardized testing score guidance was not included on many program websites. These criteria are especially important for applicants who have historically applied to more residency programs and had lower match rates, such as osteopathic medical school and international medical graduate applicants [3,13]. About half of the programs did not include information about alumni trajectories, which can be valuable for applicants trying to determine whether their professional goals around practice setting or fellowship align with those of prior residents. Program signaling presents an exciting opportunity for equity, but it is important for applicants to have the opportunity to send signals to programs that will consider their applications and align with their goals.

Improving transparency could also reduce residency programs’ burden of reviewing large volumes of applications. By describing more criteria on websites, programs could communicate which applications will be considered—before applicants have spent resources on applying or signaling. In the National Resident Matching Program’s Program Director Survey results, OBGYN residency program directors reported that an average of over 45% of applications are rejected based on standardized screening tools, before holistic review [14]. Failing to transparently describe criteria for standard screening tools can perpetuate rising application numbers and costs if applicants unknowingly apply to programs where their applications are automatically screened out of consideration.

**Figure 1.**
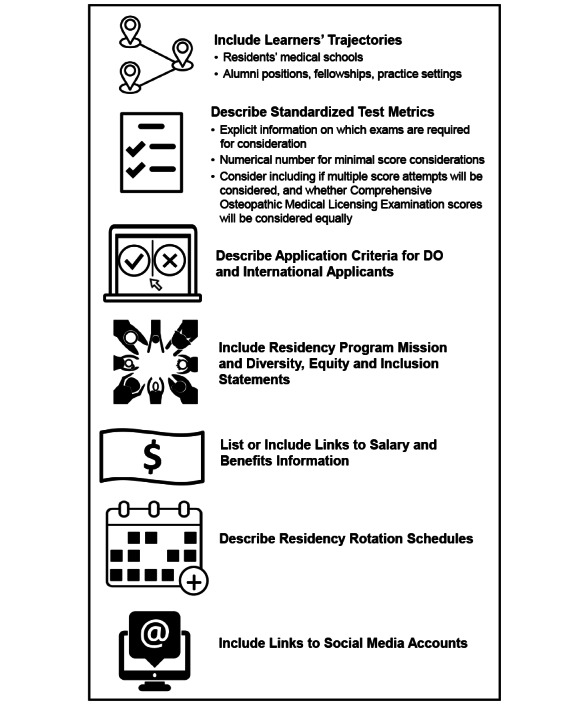
Best practices of what should be included in obstetrics and gynecology residency program websites. DO: doctor of osteopathic medicine.

From this work, we propose best practices for residency program websites in Figure 1. The practices are informed by the authors’ perspectives as applicant, resident, and OBGYN faculty stakeholders in the residency application process. These practices include describing transparent application criteria to help applicants understand if they qualify for consideration, statements about values and outcomes that illuminate program priorities, and logistic considerations that can influence whether a program is a feasible option for an applicant. If applicants have access to this information, they may identify a more targeted list of programs to which they can apply and send signals, which will ultimately aid in improving the residency application process for applicants and programs alike.

The US residency application process needs multiple reforms to improve match rates and increase favorable outcomes for applicants [3]. Signaling may prove to be an important component of this reform, but signaling can only be successful if applicants can send informed signals to programs that align with their goals and values. One opportunity for residency programs to contribute to the success of this reform is sharing information, such as our residency website best practices, that help applicants determine whether the program aligns with their qualifications, desires, and goals.

### Limitations

Some programs may not control their website content; instead, they may follow graduate medical education or organization-specified templates. Nevertheless, our work provides important information for these groups to make choices about website content and we propose best practices to consider in Figure 1.

In this study, we collected data regarding USMLE Step 1 and COMLEX Level 1 examination scores. However, both exams have transitioned to a pass-or-fail grading system—USMLE Step 1 in January 2022 and COMLEX Level 1 in May 2022. Therefore, our data regarding USMLE Step 1 and COMLEX Level 1 scores may not apply to future applicants. Effects of a pass or fail grading system in the application process are yet to be determined, but other criteria, such as USMLE Step 2 and COMLEX Level 2 scores, may take on increasing importance. Websites must be updated to accurately reflect program requirements, so we suggest this is an excellent opportunity to provide increased information to applicants, such as clearly stating testing requirements, whether multiple attempts at exams are accepted, and if there are USMLE Step 2 or COMLEX Level 2 score thresholds or guidelines for applicants.

### Comparison With Prior Work

This work aligns with findings in other specialties and illustrates key findings that will be of value given the evolving state of residency application processes. OBGYN programs’ websites had rates of listing residents’ medical schools, salary, benefits, and rotation schedules that are similar to those of other specialties [15-20]. Application selection criteria were more difficult to compare because definitions varied across studies. However, like several other specialties, less than half of OBGYN residency programs included specific USMLE Step 1 score guidance [15-18,21]. Additionally, OBGYN programs, like several other specialties, do not universally indicate whether programs consider international medical graduate students and can sponsor visas [20,21]. However, some OBGYN program websites do stand out for including diversity, equity, and inclusion information and case numbers more often than some other specialties [16,22].

Our comparison of different types of programs is less common. Studies in two other specialties compared academic and non-academic programs and found academic programs included more of the characteristics they studied, which aligns with our findings in OBGYN [20,22]. Given the inherent value and differences in all programs, we believe that comparing types of residency programs presents an opportunity to understand which programs can improve in communicating with applicants.

### Conclusions

In this competitive application landscape, it is crucial that applicants are provided equitable access to information that allows them to determine where to apply and send signals to optimize their success in matching at a program aligned with their values. Applicants use websites to determine residency program qualities, but the onus of deciphering the best fit should not rest entirely on them. A robust presentation of residency program personnel, curriculum, values, benefits, and application criteria can help applicants understand where their applications will be considered, and possibly where their signals are most strategic. Increased information sharing on program websites could contribute to an improved application process.

## Abbreviations

COMLEX: Comprehensive Osteopathic Medical Licensing Examination
DO: doctor of osteopathic medicine
ERAS: Electronic Residency Application Service
MD: medical doctorate
OBGYN: obstetrics and gynecology
USMLE: United States Medical Licensing Examination
